# Editorial: Look who’s talking: Dialogues with beta cells

**DOI:** 10.3389/fendo.2022.1117181

**Published:** 2023-01-05

**Authors:** Noèlia Téllez, Anabel Rojas, Rosa Gasa

**Affiliations:** ^1^ Facultat de Medicina, Universitat de Vic – Universitat Central de Catalunya (UVic-UCC), Barcelona, Spain; ^2^ Institut de Recerca i Innovació de la Catalunya Central (IRIS-CC), Barcelona, Spain; ^3^ Centro Andaluz de Biología Molecular y Medicina Regenerativa (CABIMER) - Universidad Pablo de Olavide-Universidad de Sevilla, Consejo Superior de Investigaciones Científicas (CSIC), Seville, Spain; ^4^ Centro de Investigación Biomédica en Red de Diabetes y Enfermedades Metabólicas Asociadas (CIBERDEM), Madrid, Spain; ^5^ Institut d'Investigacions Biomèdiques August Pi i Sunyer (IDIBAPS), Madrid, Spain

**Keywords:** beta cell, crosstalk, communication, diabetes, islet

The lives of cells are not solitary. Important elements of cell biology, such as function, differentiation, proliferation, and survival, are regulated through communication between cells located in separate organs and between cells of the same kind or distinct types within a tissue.

Pancreatic beta cells are regularly in communication with multiple cell types located within the islets of Langerhans as well as with cells outside the islets including cells of the exocrine pancreas, liver, muscle, adipose tissue, brain, or gut. These connections involve a variety of chemical and mechanical signals, are highly dynamic, and can tailor the behavior of beta cells in the short and long-term, ultimately contributing to the control of glucose homeostasis. Importantly, the types of communication and messages that they convey can change throughout the life of the organism. Thus, certain cell dialogues only take place during specific windows of time, i.e. embryogenesis or pregnancy, and mediate beta cell development and expansion under these physiological situations. On the other hand, some cell dialogues are forced upon pathological situations, such in the case of type 1 diabetes *via* the arrival of unexpected new neighbors of the immune system, or in the case of obesity *via* the reception of an unprecedented volume of signals from enlarged fat tissue. These latter dialogues often end in a difficult to resolve conflict that leads to beta cell dysfunction and/or loss and the onset of diabetes. A plethora of mouse models and cell lines have been generated in order to unravel the connection between pancreatic beta cells and other cells from various tissues aimed to identify key targets for diabetes treatment. Still, the identification of many mediators in such connections remains a challenge.

The reviews by Langlois et al. and Overton et al. emphasize some of the close connections made inside the islets between beta cells and their neighbors. This intricate communication network is reflected in the first instance by the hierarchical network between the beta cells themselves, where hub cells wire the rest of the beta cells for the proper secretion of insulin. Correct glucose homeostasis also depends on the interactions between the beta cells and alpha cells, in which the reciprocal signals between both cell types through the secretion of metabolites reflect genuine cellular cooperation rather than antagonism. The communication between beta and alpha cells can acquire a higher level of complexity by the action of third parties, such as δ-cell secreted somatostatin or ε-cell secreted ghrelin and obestatin, or even other intermediate cells, like intestinal cells.

The exocrine pancreas, where the islets are scattered in, also plays a crucial function in maintaining glucose homeostasis by signaling to beta cells. Overton et al. explore pancreatic cellular crosstalk by examining the developmental cues that can instruct and manipulate certain pancreatic lineage decisions, the role of intra-islet communication and extracellular connections in beta cell function, and how exocrine pancreas function and dysfunction can influence the endocrine pancreas.

The reviews of Langlois et al., López-Bermudo et al., and Martínez-Montoro et al. highlight long-distance communication between beta cells and extra-pancreatic organs and include several examples of the mechanisms mediating these organ-to-organ communications. The crosstalk between the liver and pancreas plays an essential role in glucose and lipid metabolism and is central to accommodate to changing nutritional states. López-Bermudo et al. review well-known and novel circulating factors, released either by hepatocytes or by beta cells, which could contribute to this adaptation by modulating pancreatic and hepatic function. When it comes to beta cell proliferation and survival, cytokines, hepatokines and myokines released either by the liver or by the skeletal muscle can have either negative or positive effects, depending on the insulin-resistance state of these tissues. Much is unknown about the skeletal muscle secretome and how variations in this affect beta cell mass in diabetes.

Due to the well-known connection between type 2 diabetes and obesity, adipose tissue is in a prime position to trigger beta cell malfunction. In this line, adipokines, pro-inflammatory signals and free fatty acids released by the adipose tissue have been associated with beta cell damage and functional impairment. But beta cells are not only helpless victims; through persistent hyperinsulinemia, they can also promote fibrosis, senescence, and inflammation in adipose tissue, creating a vicious cycle that has a negative influence on metabolic control. Another important participant in this intricate communication network is the gut microbiome. Martínez-Montoro et al. exposes how pathological alterations to the gut microbiota can affect inflammation of the adipose tissue, fat deposition, and adipokine balance, and these in turn may have an impact on beta cell survival and function. In addition, although the mechanisms underlying these effects remain to be comprehensively elucidated, gut microbiota and related substances may directly influence beta cell development and insulin secretion.

## Conclusion

Knowledge of the cellular connections within the pancreas or between the pancreas and other tissues in both health and disease will allow for a better understanding of the complexities of disease progression as well as the identification of potential cellular targets or mediators that could be modulated to delay, ameliorate, or even reverse pancreatic dysfunction.

## Author contributions

All authors listed have made a substantial, direct, and intellectual contribution to the work and approved it for publication.

